# Supplementary material for: Differential Effects of Nonsteroidal Anti-Inflammatory Drugs in an In Vitro Model of Human Leaky Gut
Source: Cells. 2023 Feb 24;12(5):728. doi: 10.3390/cells12050728 (PMC10001324; doi:10.3390/cells12050728)
Supplement: Supplementary file 1 [file cells-12-00728-s001.zip › cells-2128031-supplementary.pdf]

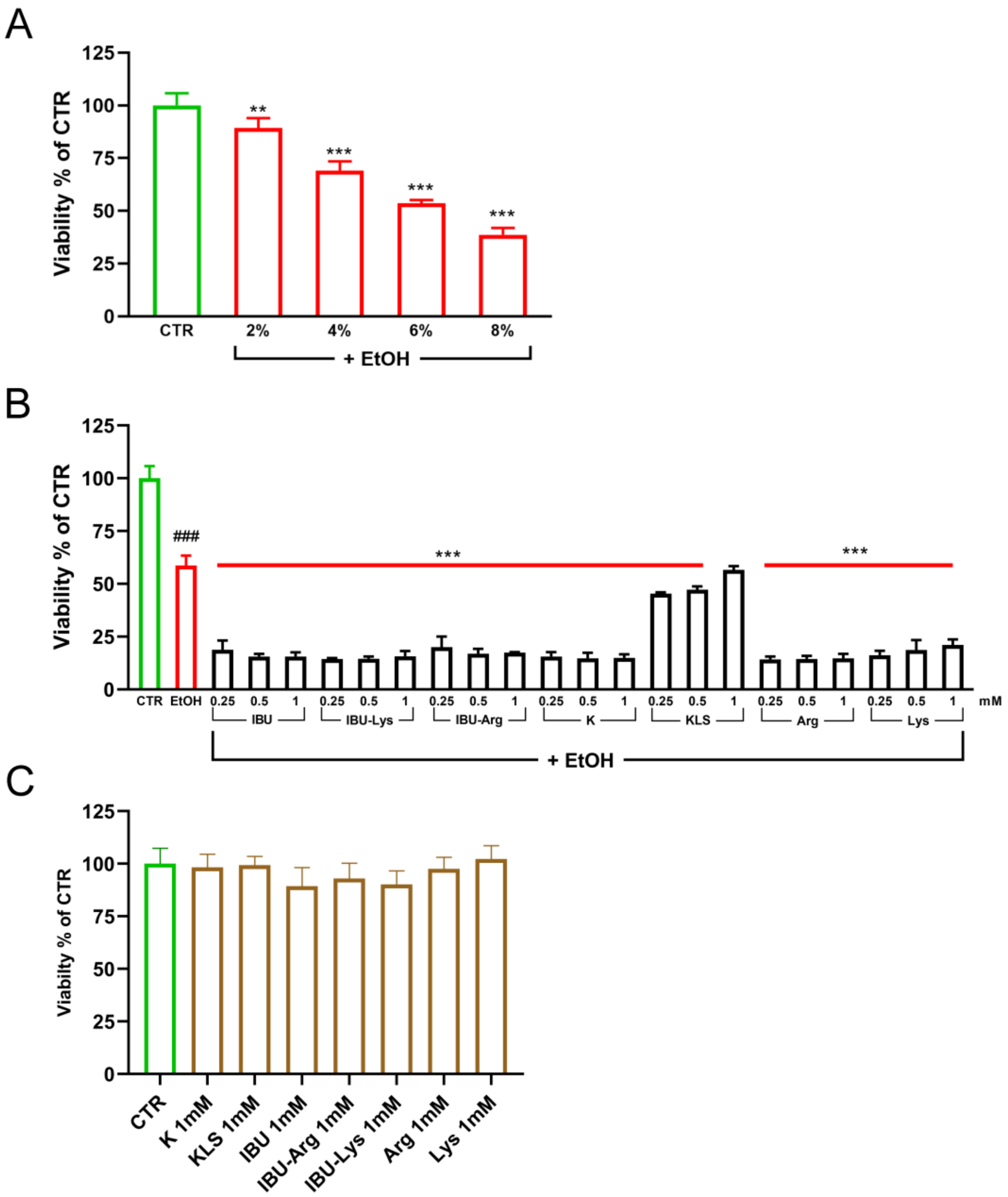

Figure S1: **(A)** Cell viability assay for Caco-2 upon different concentrations of EtOH. **,p< 0.005; ***,p<0.0001 vs CTR. **(B)** Cell viability assay for Caco-2 upon EtOH 6% and the tested NSAIDS Arg and Lys alone treated for 72h.***,p<0.0001 vs ETOH; ###, p<0.0001 vs CTR. **(C)** Cell viability assay for Caco-2 upon the tested NSAIDs and Arg and Lys alone treated for 72h.
